# First-line systemic treatment for hepatocellular carcinoma: A systematic review and network meta-analysis

**DOI:** 10.1016/j.heliyon.2023.e18696

**Published:** 2023-07-26

**Authors:** Domenico Ciliberto, Giulio Caridà, Nicoletta Staropoli, Caterina Romeo, Grazia Maria Arillotta, Cristina Napoli, Luigia Gervasi, Francesco Luciano, Caterina Riillo, Pierfrancesco Tassone, Pierosandro Tagliaferri

**Affiliations:** a Renato Dulbecco Hospital, Catanzaro, Italy; b Department of Experimental and Clinical Medicine, Magna Græcia University, Catanzaro, Italy; c Sbarro Institute for Cancer Research and Molecular Medicine, Center for Biotechnology, College of Science and Technology, Temple University, Philadelphia, PA, USA

**Keywords:** HCC, Advanced hepatocellular carcinoma, TKI, ICI, Sorafenib, Lenvatinib, Atezolizumab, Durvalumab, Tremelimumab, First line

## Abstract

The rapid development of novel therapeutic options for advanced hepatocellular carcinoma (aHCC) has generated some uncertainty about the rational choice of the systemic upfront treatment. So far, a variety of therapeutic strategies have been investigated, including the combination of immunecheckpoint inhibitors and *anti*-VEGF. To identify the treatment that should be preferred as front-line approach, we compared the efficacy and toxicity of a variety of therapeutic strategies. With this aim, we performed a systematic review and a meta-analysis of randomized clinical trials. OS, PFS, ORR and tolerability outcomes were considered, and for each outcome the treatment ranking was evaluated by the surface under the cumulative rankings (SUCRAs). Combination of Camrelizumab + Rivoceranib scored the best in OS, followed by Sintilimab + Bevacizumab, whereas Lenvatinib + Pembrolizumab showed higher probability to be the best treatment in PFS and Sintilimab + Bevacizumab performed best in ORR. Finally, Durvalumab is the most tolerated treatment.

## Graphical abstract

**Figure:**
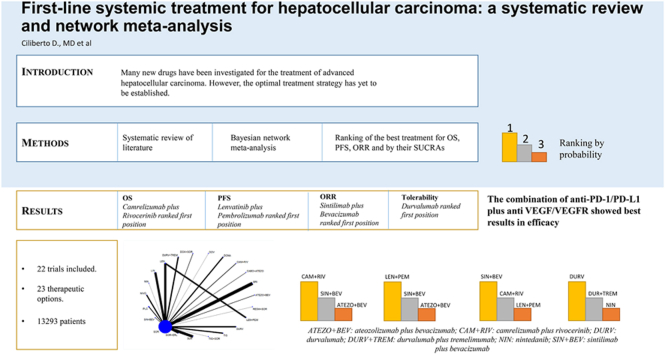

## 1 Introduction

Hepatocellular carcinoma (HCC) is the sixth most common cancer worldwide [1] and carries a severe prognosis [2]. The incidence and mortality of HCC are increasing in Europe and USA [3]. Epidemiologically, men are at a higher risk of developing liver cancer compared to women. Although since 2008 new therapies emerged for the advanced HCC (aHCC), such as Sorafenib, a tyrosine kinase inhibitor (TKI), HCC remains a poor prognosis disease [4]. After the SHARP trial, other small molecules showed a non-inferiority efficacy compared to Sorafenib, such as Lenvatinib but only the use of immunotherapy opened a new clinical era. At the present the best treatment option for untreated aHCC is unclear, and a sequencing strategy for second-third line therapies needs to be investigated. Furthermore, many new drugs have been investigated in clinical trials, but direct comparisons are never available for all potential schedules. For this reason, the aim of this study is to compare the efficacy and toxicity of new therapeutic strategies for the front-line treatment of aHCC, by a systematic review and network meta-analysis (NMA), which allows direct, indirect and mixed comparisons in order to provide a ranking of probabilities of all schedules. The final goal is to provide a comprehensive view of the current treatment landscape with the aim to help decision-making and rationally design treatment sequences.

## 2 Methods

In the time-frame from 2008 (in which Sorafenib was identified as the first systemic treatment after the results of SHARP trial) to September 2022, according to the Preferred Reporting Items for Systematic Review and Meta-Analyses (PRISMA) statement [5], a comprehensive search from available online (PubMed, Embase, MedLine, Cochrane Central Register) and meeting (ASCO and ESMO) databases was performed. Search methods were performed are detailed in Supplementary S1. Two investigators (GC and DC) independently performed the database consultation and records selection, and disagreements were solved after consensus. The risk of bias was evaluated using the modified Cochrane Collaboration tool to assess risk of bias for randomized controlled trials, that considers: random sequence generation (selection bias), allocation concealment (selection bias), blinding of participants and researchers, blinding of outcome assessment (detection bias), incomplete outcome data (attrition bias), selective reporting (reporting bias) [6].

The outcomes considered were: Overall Survival (OS), Progression Free Survival (PFS), Objective Response Rate (ORR) and tolerability. For OS and PFS we extracted the hazard ratios (HR) and relative Confidence Intervals (CIs). The response was defined as the sum of absolute complete responses (CR) and partial responses (PR) according to RECIST 1.1, whereas the toxicity was reported as the sum of patients who experienced AEs of grade ≥3. For ORR, the Odds Ratios were calculated and compared, whereas for toxicity Risk Ratios were calculated and compared.

The eligibility criteria for study inclusion are listed as follow: randomized controlled trials, systemic first line treatment for aHCC with TKIs, immune-check-point inhibitors (ICIs), *anti*-VEGF or their combination. Prior intervention procedures such as TACE were allowed.

The exclusion criteria were: not randomized trials, RCTs with no published results, trials with a retrospective comparator, studies with unclear methodology, trials including previously treated patients with systemic drugs, treatment compared to chemotherapy strategies only, non-English papers. Inclusion and exclusion criteria based on PICOS model are reported in Supplementary S2. The risk of publication bias was assessed by visual inspection of Funnel plot. Due to different therapeutic strategies lacking direct comparison, a Bayesian Network Meta-Analysis (NMA) was performed [7,8]. The analysis was carried out using STATA software (Stata-Corp, version 17) [9] implemented by a graphical tool and the *mvmeta* package [10]. For each outcome of interest, a Bayesian NMA was performed using a Markov-Chain Monte Carlo [11] simulation technique with up to 30,000 iterations. The network plot was built through the command *networkplot* [12]. The NMA was performed using the commands *network meta c*^12^, and the best treatment for each outcome was assessed thought *netrank* and *sucra* commands [13]. The surface under the cumulative ranking curve (SUCRA), which provides a numerical summary of the rank distribution of each treatment schedule on the different endpoints, provided hierarchy. In order to obtain a square matrix reassuming the effect of each treatment, we used the command *netleague*. In the leagues, the outcomes are reported with corresponding 95% credible intervals (CrIs), as expected for a Bayesian Meta-Analysis (NMA) [14]. The league-tables show information about the estimated summary effect for each comparison. More is the summary effect closer to 1, lower is the difference between two treatments: if the CrI contains 1, the difference is not statistically significant [7,15].

For each outcome of interest (OS, PFS, ORR and tolerability), a NMA was performed. If a trial was deficient in some information about an outcome (e.g., HR for OS, detailed incidence of SAE or ORR), it was excluded from the NMA of that outcome.

Finally, treatments were compared in a bidimensional plot using SUCRAS for OS and tolerability through the command *clusterank*, and an heatmap was created to compare all other outcomes using GraphPad PRISM 9.

## 3 Results

### 3.1 Search results

**Fig. 1:**
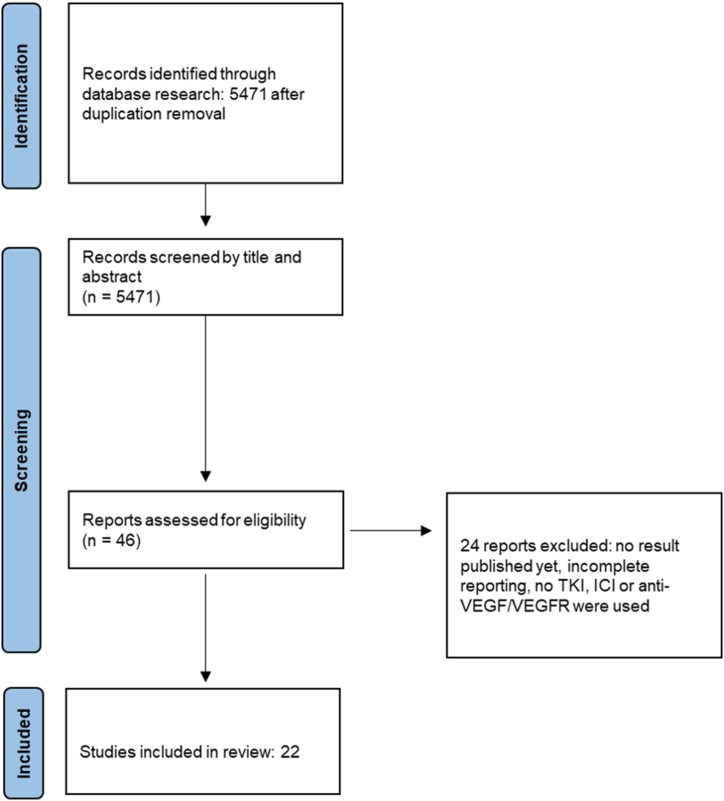
– PRISMA chart. Flow chart of the results by systematic review of the main databases according to PRISMA.

After duplications removal, a total of 5471 record were evaluated, and 22 RCTs were assessed for the quantitative analysis (Fig. 1), for a total of 13293 patients. The characteristics of the included RCTs ([4,[16], [17], [18], [19], [20], [21], [22], [23], [24], [25], [26], [27], [28], [29], [30], [31], [32], [33], [34], [35]],[36]) are detailed in Table 1. Included treatment regimens were Atezolizumab *plus* Bevacizumab (ATEZO + BEV), Brivanib (BRI), Cabozantinib *plus* Atezolizumab (CABO + ATEZO), Camrelizumab *plus* Rivoceranib (CAM + RIV), Donafenib (DONA), Dovitinib (DOV), Doxorubin *plus* Sorafenib (DOX + SOR), Durvalumab (DURV), Durvalumab *plus* Tremelimumab (DURV + TREM), GEMOX *plus* Sorafenib (GEMOX + SOR), Lenvatinib (LEN), Lenvatinib *plus* Pembrolizumab (LEN + PEM), Linifanib (LIN), Nintedanib (NIN), Nivolumab (NIVO), Placebo (PLC), Resminostat + Sorafenib (RESM + SOR), Sintilimab *plus* Bevacizumab (SIN + BEV), Sorafenib (SOR), Sorafenib *plus* Erlotinib (SOR + ERL), Sunitinib (SUN), Tislelizumab (TIS), Tigatuzumab + Sorafenib (TIG + SOR). In none of the NMA performed for each outcome triangular or quadratic loops were found.

**Table 1:** Clinical trials included.

Author	Year	Experimental arm	Control arm	Randomization	Sample size	Trial type	Region	Median OS and PFS (months)	HR OS and PFS	Number of responses
Llovet et al. (SHARP Trial)	2008	Sorafenib	Placebo	1:1	601	Phase III	Western regions	**OS**: 10.7 vs 7.9 **PFS**: not performed	**OS**: 0.69 (CI, 0.55–0.87) **PFS**: not performed	**Experimental**: 7 **Control**: 2
Cheng et al. (Asian SHARP)	2009	Sorafenib	Placebo	2:1	226	Phase III	Asia	**OS**: 6.5 vs 4.2 **PFS**: not performed	**OS**: 0.68 (CI, 0.50–0.93) **PFS**: not performed	**Experimental**: 5 **Control**: 1
Johnson et al. (BRISK-FL)	2013	Brivanib	Sorafenib	1:1	1155	Phase III	Global	**OS**: 9.9 vs 9.5 **PFS**: not performed	**OS**: 1.06 (CI, 0.93–1.22) **PFS**: not performed	**Experimental**: 69 **Control**: 51
Cheng et al.	2013	Sunitinib	Sorafenib	1:1	1074	Phase III	Asia	**OS**: 7.9 vs 10.2 **PFS**: 3.6 v 3.0	**OS**: 1.30 (CI, 1.13–1.5) **PFS**: 1.13 (CI: 0,99–1,3)	**Experimental**: 35 **Control**: 33
Zhu et al. (SEARCH)	2015	Sorafenib plus erlotinib	Sorafenib plus placebo	1:1	720	Phase III	Global	**OS**: 9.5 v 8.5 **PFS**: not available	**OS**: 0.93 (CI, 0.78–1.11) **PFS**: 1.11 (CI, 0.94–1.31)	**Experimental**: 24 **Control**: 14
Cainap et al.	2015	Linifanib	Sorafenib	1:1	1035	Phase III	Global	**OS**: 9.1 vs 9.8 **PFS**: 4.2 vs 2.9	**OS**: 1.05 (CI, 0.9–1.22) **PFS**: 0,81 (CI, 0.7–0,95)	**Experimental**: 67 **Control**: 36
Cheng et al.	2015	Tigatuzumab plus Sorafenib	Sorafenib	1:1:1	162	Phase II	Europe, United States	**OS**: 12.2* vs 8.2 **PFS**: not performed	**OS**: 1,04 (CI, 0,64 1,67) **PFS**: not performed	**Experimental**: not performed **Control**: not performed
Tak et al.	2015	Resminostat + Sorafenib	Sorafenib	1:1	167	Phase I/II	Asia	**OS**:14.1 vs 11.8 **PFS**: not performed	**OS**:1.04 (CI, 0.704–1.555) **PFS**: not performed	**Experimental**: 3 **Control**: 8
Cheng et al.	2016	Dovitinib	Sorafenib	1:1	165	Phase II	Asia	**OS**: 34.6 vs 36.7 **PFS**: not performed	**OS**: 1.27 (CI, 0.90–1.79) **PFS**: not performed	**Experimental**: 6 **Control**: 10
Kudo et al. (Reflect trial)	2018	Lenvatinib	Sorafenib	1:1	954	Phase III	Global	**OS**: 13.6 vs 12.3 **PFS**: 7.4 vs 3.7	**OS**: 0.92 (CI, 0.79–1.06) **PFS**: 0.66 (CI, 0.57–0.77)	**Experimental**: 90 **Control**: 31
Palmer et al.	2018	Nintedanib	Sorafenib	2:1	93	Phase I/II	Asia	**OS**: 11.9 vs 11.4 **PFS**: 5.3 vs 3.9	**OS**: 0.88 (CI, 0.52–1.47) **PFS**: 1.35 (CI, 0.78–2.34)	**Experimental**: 7 **Control**: 6
Abou-Alfa et al. (CALGB 80802)	2019	Doxorubicin plus Sorafenib	Sorafenib	1:1	356	Phase III	Global	**OS**: 9.3 vs 9.4 **PFS**: 4.0 vs 3.7	**OS**: 1.05 (CI, 0.83–1.31) **PFS**: 0.93 (CI, 0.75–1.16)	**Experimental**: 15 **Control**: 8
Assenat et al.	2019	GEMOX plus Sorafenib	Sorafenib	1:1	83	Phase II	Global	**OS**: 13.5 vs 14.8 **PFS**: 6.2 vs 4.6	**OS**: not performed **PFS**: not performed	**Experimental**: 6 **Control**: 4
Yau et al. (CheckMate-459)	2019	Nivolumab	Sorafenib	1:1	743	Phase III	Global	**OS**: 16.4 vs 14.7 **PFS**: 3.7 vs 3.8	**OS**: 0.85 (CI, 0.72–1.02) **PFS**: not available	**Experimental**: 57 **Control**: 26
Finn et al. (IMbrave-150)	2020	Atezolizumab plus Bevacizumab	Sorafenib	2:1	501	Phase III	Global	**OS**: NE vs 13.2 **PFS**: 6.8 vs 4.3	**OS**: 0.66 (CI, 0.52–0.85) **PFS**: 0.59 (CI, 0.47–0.76)	**Experimental**: 89 **Control**: 19
Bi et al.	2020	Donafenib	Sorafenib	1:1	668	Phase III	Asia	**OS**: 12.1 vs 10.3 **PFS**: 3.7 v 3.6	**OS**: 0.83 (CI, 0.71–0.99) **PFS**: 0.91 (CI, 0.76–1.08)	**Experimental**: 20 **Control**: 12
R. K. Kelley et al. (Cosmic-312)	2021	Cabozantinib plus Atezolizumab	Sorafenib	2:1:1	837	Phase III	Global	**OS**: not available **PFS**: 6.8 vs 4.2	**OS**: 0.90 (CI, 0.69–1.18) **PFS**: 0.63 (CI, 0.44–0.91)	**Experimental**: 27 **Control**: 5
Ren Z. et al. (Orient-32 Trial) [32]	2021	Sintilimab plus bevacizumab	Sorafenib	2:1	571	Phase III	Asia	**OS**: NR vs 10.4 **PFS**: 4.6 vs 2.8	**OS**: 0·57 (CI, 0·43–0·75) **PFS**: 0·56 (CI, 0·46–0·70)	**Experimental**: 75 **Control**: 7
Abou-Alfa et al. (Himalaya Trial) [37]	2022	Durvalumab plus Tremelimumab/Durvalumab	Sorafenib	1:1:1	1171	Phase III	Global	**OS** (Durvalumab plus Tremelimumab): 16.4 vs 13.8 **OS** (Durvalumab): 16.6 vs 13.8 **PFS** (Durvalumab plus Tremelimumab): 3.8 vs 4.1 **PFS** (Durvalumab): 3.7 vs 4.1	**OS** (Durvalumab plus Tremelimumab): 0.78; CI, 0.65–0.92 **OS** (Durvalumab): 0.86; CI, 0.73–1.03 **PFS** (Durvalumab plus Tremelimumab): 0.9; CI, 0.77–1.05. **PFS** (Durvalumab): 1.02; CI, 0.88–1.19	**Experimental** (Durvalumab plus Tremelimumab): 79 **Experimental** (Durvalumab): 66 **Control**: 19
Finn RS et al. (LEAP-002)	2022	Lenvatinib plus Pembrolizumab	Lenvatinib	1:1	794	Phase III	Global	**OS**: 21.2 vs 19.0 **PFS**:8.2 vs 8.1	**OS**: 0.84 (CI, 0.81–0.99) **PFS**: (CI, 0.83–0.98)	**Experimental**: 103 **Control**: 70
Kudo et al. (Rationale-301)	2022	Tislelizumab	Sorafenib	1:1	674	Phase III	Asia	**OS**: 15.9 vs 14.1 **PFS**: not performed	**OS**: 0,85 (CI, 0,71–1,01) **PFS**: not performed	**Experimental**: 49 **Control**:19
Qin S. et al. (SHR-1210-III-325)	2022	Carmelizumab plus rivoceranib	Sorafenib	1:1	543	Phase III	Asia	**OS**: 22.1 vs 15.2 **PFS**: 5.6 vs 3.7	**OS**: 0,52 (CI, 0,41–0,65) **PFS**: 0,62 (CI, 0,49–0,8)	**Experimental**: 69 **Control**:19

Legend: OS: Overall Survival, PFS: Progression Free Survival, CI: Confidence Interval. *Tigatuzumab was administered at 6/6 mg/kg and 6/2 mg/kg. Median OS was 12.2 month in patients who received Tigatuzumab 6/6 mg/kg and 8.2 month in patients who received Tigatuzumab 6/2 mg/kg.

For all trials a full article was available, except for Finn RS et al. (LEAP-002), Kudo et al. (Rationale-301) et Qin S. et al. (SHR-1210-III-325), that were recently presented at ESMO 2022. Five RCTs were phase II, phase Ib/II or phase II-III. In all trials only patients with Child-Pugh of class A were enrolled, for the exception for phase Ib of phase II trials in which Child-Pugh B patients were admitted for the non-randomized phase, but not for randomized part. In all trials, performance status according to Eastern Cooperative Oncology Group score (PS ECOG) of patients was inferior to 2. Trials in which Lenvatinb was evaluated, patients witch vascular invasion greater the 50% were excluded, whereas in trial in which ICIs were evaluated patients that undergo to orthotopic liver transplantation (OLT) were excluded. However, these kinds of patients were numerically not significant and should not interfere with the comparability in the network. In 8 trials PFS was not evaluated or Time to Progression (TTP) was preferred to PFS as surrogate endpoint of efficacy. In our NMA, TTP was not used in substitution of PFS. In all trials, prior intervention procedures for localized HCC were admitted.

### 3.2 Risk of bias

The risk of bias assessment revealed a low risk of bias for all trials included in this NMA. A detailed risk of bias assessment can be found in Supplementary S3 and S4.

#### 3.2.1 Inconsistency and publication bias

No triangular or quadratic loops for evaluation of inconsistency were found for this network meta-analysis using command *ifplot*. Funnel plot visual inspection didn't show publication bias (Supplementary S5).

### 3.3 OS analysis

**Fig. 2:**
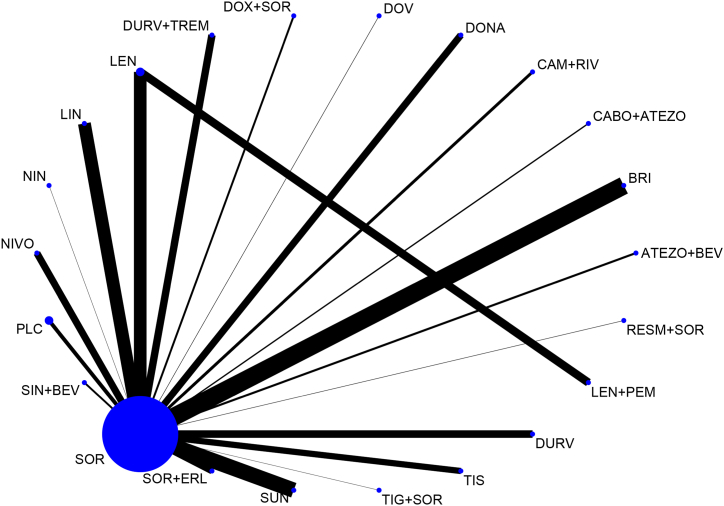
– Network plot for OS NMA. The image shows the direct comparisons of the network meta-analysis. Each circular node represents a treatment: the larger is the circle, the greater is the number of RCTs in which that treatment was investigated. The thickness of the lines is proportional to the number of patients involved in each comparison.

The NMA for OS included 21 RCTs, 13134 patients and 22 treatment options (Fig. 2). Assenat et al. trial was excluded from analysis because not data about OS was available.

**Fig. 3:**
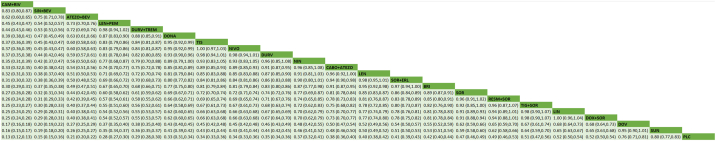
– Netleague for OS. This figure shows the estimated summary effect for each comparison in term of HR and relative 95% CrI. The treatments are sorted from the best to the worst according to SUCRAs values. HRs lower than 1 favour the first treatment. If the CrIs contains 1, the result is not statistically signifcant

CAM + RIV scored the best result (SUCRA 100%, Probably Best 100%), followed by SIN + BEV (SUCRA 95.2%), ATEZO + BEV (SUCRA 90.5%), LEN + PEM (SUCRA 84.8%) and DURV + TREM (SUCRA 85.7%). Comparing the relative hazard ratios, CAM + RIV showed a statistically significant superiority in the network in all diagonal comparisons (Fig. 3). Interestingly, the difference between Nivolumab and Durvalumab is not statistically significant 0.98 (CrI 95%, 0.94–1.01), whereas LEN won the confront with SOR, with a HR of 0.85 (CrI 95% 0.83, 0.87). Of note, the first 10 best treatments for OS are represented by ICIs or their combination with *anti*-VEGF/VEGFR, excluding Donafenib that scored the 6th position in the ranking and Nintedanib that scored the 10th position.

### 3.4 PFS analysis

Only 13 trials of the 22 included could be compared for PFS, due to the lack of HR PFS in the remaining RCTs, for a total of 8454 patients and 14 treatments (Supplementary S6). Johnson et al. (BRISK-FL), Bi et al., Cheng et al. (2016), Assenat et al., Yau et al. (CheckMate-459), Kudo et al. (2015), Zhu et al. (SEARCH), Cheng et al. (2015), Kudo et al. (Rationale-301) were excluded from this analysis because PFS was not an endpoint.

In this scenario, LEN + PEM scored the best result (SUCRA 100%, probably best 100%), whereas the second position was achieved by SIN + BEV (SUCRA 92.3%) followed by ATEZO + BEV (SUCRA 84.6%) and CAM + RIV (SUCRA 76.3%). In the pairwise comparison, LEN + PEM showed a statistically significant benefit in all diagonal comparisons, scoring a HR of 0.96 (CrI 95%, 0.93, 0.98) versus the second-best SIN + BEV (Supplementary S7).

### 3.5 ORR analysis

For the ORR analysis 21 RCTs, 13055 patients and 22 treatment options were included (Supplementary S8). Cheng et al. (2015) was excluded because no data for ORR was available.

SIN + BEV scored the first position (SUCRA 95.9%, Probably best 50.6%), followed by CAM + RIV (SUCRA 91.2%, probably best 18.5%), LEN + PEM (89.2 probably best 20.5%), DURV + TREM (SUCRA 87.9%, probably best 8.6%), DURV (SUCRA 80.2%, probably best 1.5%).

The combination therapy SIN + BEV has not showed a statistically significant difference versus CAM + RIV (Odds ratio 1.42, CrI 95%, 0.41,4.98), versus LEN + PEM (Odds Ratio 1.59, CrI 95% 0.43–7.36), versus DURV + TREM (Odds Ratio 1.74, CrI 95%, 0.50–6.08) and versus DURV (Odds Ratio 2.64, CrI 95%, 0.78–9.22). The same combination therapy showed a statistically significant difference in the remaining comparisons (Supplementary S9).

### 3.6 Tolerability analysis

The tolerability of each treatment in NMA was also compared, including 21 RCTs, 12927 patients and 22 treatment options (Supplementary S10). Cheng et al. (2015) was excluded because not enough data about ORR was available.

DURV appeared the most tolerated treatment (SUCRA 99.6%, probably best 92.7%), followed by DURV + TREM (SUCRA 95.6%, probably best 7.3%). CAM + RIV, that scored the best result in OS, is the second-last in the tolerability ranking, whereas LEN + PEM, that scored the best position in PFS, ranked the 19^h^ position. CABO + ATEZ is the most toxic treatment of this network. The difference between DURV and DURV + TREM appears to be non-statistically significant (Supplementary S11).

### 3.7 Summary of SUCRAs and clusterank

**Fig. 4:**
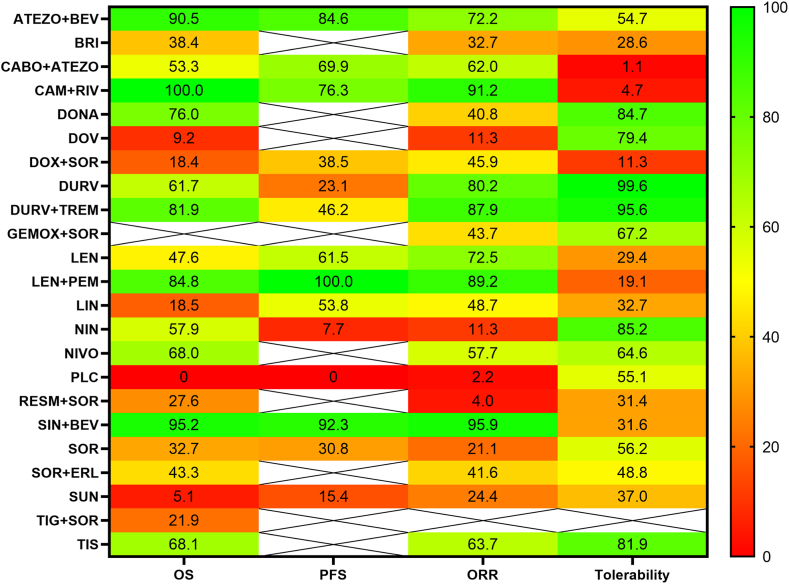
– Heatmap of SUCRAs. In + this figure are reported the SUCRA values of each treatment for each outcome. A SUCRA value close to 100% is coloured in green, whereas a SUCRA value close to 0% is coloured in red.

All treatments were compared by their SUCRAs in each outcome of interest in a grouped heatmap. (Fig. 4). Representation of SUCRAs for each outcome is reported in Supplementary (S12, S13, S14 and S15).

**Fig. 5:**
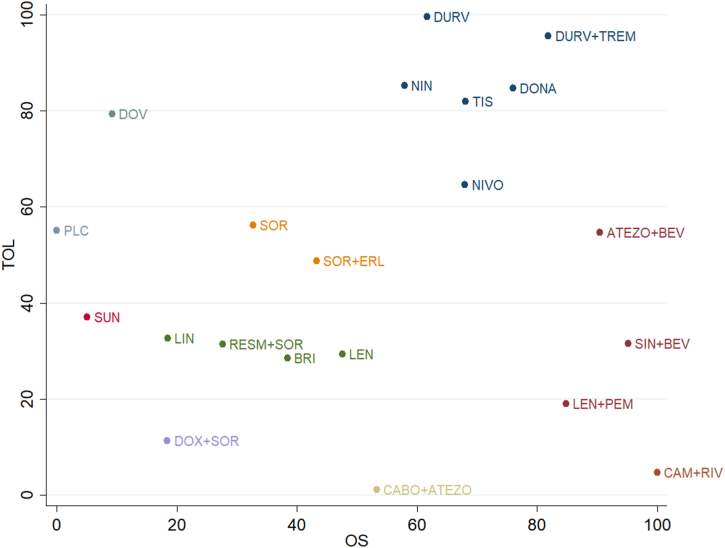
– Cluster Rank. The image reports the distribution of each treatment in a bidimensional plot, in which the X-axis represents the SUCRA value of OS from 0 to 100%, and the Y-axis represents the SUCRA value of toxicity from 0 to 100%.

Finally, OS and tolerability were reported in a bidimensional plot. Cheng. et al. (2015) trial was excluded from cluster rank because AEs data were insufficient to calculate Risk Ratio, whereas Assenat et al. trial was excluded because no HR for OS was provided (see Fig. 5).

## 4 Discussion

The selection of upfront systemic therapy for aHCC patients is challenging due to the intrinsic heterogeneity of the disease and the variety of currently available treatments. Moreover, to date, no biomarkers are available for patients’ personalized treatment. During the last few years, several effective drugs and treatment strategies were investigated, mainly TKIs (Sorafenib being the first-in-class drug) and ICIs alone or in combination with *anti*-angiogenetic agents or other ICIs.

Sorafenib was the first TKI approved for HCC [4]. Later, other TKIs were evaluated in non-inferiority or non-inferiority/superiority trials compared to Sorafenib. According to our meta-analysis, Lenvatinib, another FDA-approved TKI for the upfront treatment HCC compared to Sorafenib in a non-inferiority trial [24] scored better results in OS and ORR compared to Sorafenib, resulting also better tolerated.

More interestingly, the combination of Camrelizumab, an *anti*-PD-1 antibody, and Rivoceranib, a VEGFR2 inhibitor, scored the best in OS, followed by SIN + BEV, ATEZO + BEV, LEN + PEM and the *anti*-PD-L1/*anti*-CTLA-4 combination of DURV + TREM. This result can be ascribed to HCC and its microenvironment capabilities to suppress the immune response and to induce immune tolerance, neo angiogenesis playing a major role [38]. VEGF induces immune suppression also by inhibiting dendritic cells and impairing lymphocytes-infiltration [39]. A potential additional mechanism of immunologic escape may be increased expression of FasL on endothelial cells [40]. FasL can induce apoptosis of CD8^+^ cells sparing the Treg cells, promoting tolerance and tumor growth. This mechanism is enhanced by VEGF-A, overexpressed by HCC cell [38]. Consequently, the combination of *anti*-VEGF or *anti*-VEGFR and *anti*-PD-1/PD-L1 suppresses a potential mechanism of immunologic tolerance by inhibition of VEGF and reduction of FasL levels, hence promoting CD8^+^ tumor infiltration and the responsiveness of HCC to checkpoint inhibitors. In fact, in our meta-analysis, combination of ICI and *anti*-VEGF/VEGFR showed the best results in terms of OS, PFS, and ORR. Of note, the combination strategies are burdened by higher toxicity. In fact, CAM + RIV and CABO + ATEZO resulted in the most toxic treatments of the network. From the visual inspection of our cluster rank, ATEZO + BEV got, however, a good position comparing OS and tolerability simultaneously. In term of tolerability, ICI monotherapy like DURV, TIS and NIVO and the combination of DURV + TREM showed lower toxicity than TKI or combinatory treatments.

The combination of ICIs and *anti*-VEGF/VEGFR showed the best results in terms of efficacy for the first-line treatment of HCC. In the present NMA, Sintilimab showed better results than Atezolizumab in OS and PFS. This result could reflect the differences in patients recruited in IMbrave-150 and Orient-32, as stated by Ren Z. et al. who reported how patients recruited in Orient-32 trial were mostly of Chinese ancestry, younger, had a better performance status and were all mostly affected by HBV-infection (94%) compared to patients recruited in IMbrave-150 (trial in which only 50% of patients was affected by HBV-infection and 60% of subjects enrolled was non-Asiatic) [32]. Similar population characteristics are present in SHR-1210-III-325 trial in which Camrelizumab was investigated: only 17% of patients were non-asiatic, 75% of subjects was affected by HBV. Notably, to our analysis, some differences between *anti*-PD-1 and *anti*-PDL-1 appear in efficacy, activity, and toxicity. In fact, SIN + BEV and CAM + RIV show better performance in OS and ORR than ATEZO + BEV. Moreover, *anti*-PDL-1 Durvalumab, alone or in addition to Tremelimumamb, appears less toxic than Nivolumab and Tislelizumab in monotherapy. These differences could be explained by the different populations recruited in different trials, or alternatively these results could be biased by different procedures in trials management. Our NMA shows some possible discrepancies in terms of tolerability. These results could be biased by the different mechanism of action of the multitude of drugs simultaneously compared in this work. Indeed TKI, ICI, *anti*-VEGR and their combination have different tolerability and different profile in term of AEs type and grade. In our work, we ranked treatments in term of incidence of AEs of grade ≥3, without discriminating the type of AE. Therefore, in this scenario, Durvalumab monotherapy appears to be more tolerated than other strategies. These data on toxicity could help to find out general considerations in term of tolerability in a population unselected for risk of AE or patients characteristics, but limited information on different drugs and trials cannot allow unbiased ranking of most agents.

Another combination therapy evaluated in this NMA is CABO + ATEZO [31]. This combination showed interesting results for the treatment of advanced renal cell carcinoma [41] and metastatic castration-resistant prostate cancer [42], supporting the combination of TKIs and *anti*-PDL-1. In fact, Cabozantinib inhibits VEGFR and may modulate the microenvironment of HCC, reducing immunosuppression, recruiting cytotoxic T lymphocytes and enhancing response to ICIs. However, the combination of TKIs *plus* ICIs could be burdened by high toxicity, as demonstrated in our analysis, and in COSMIC-312 trial the combination CABO-ATEZO didn't shown a clinically significant increase of OS. Considering the low efficacy and the high toxicity, the combination of CABO + ATEZO appears not to be of high priority for the clinical practice, whereas, at the present, ATEZO + BEVA is approved and used in many countries.

The preliminary results of the Himalaya trial were recently presented at ASCO GI 2022. In this phase III RCT combination based on two check-point inhibitors, DURV + TREM, showed a significant benefit in terms of OS compared to Sorafenib, and durvalumab monotherapy was non-inferior to Sorafenib. Based on our analysis, this new combination DURV + TREM seems highly effective in term of ORR. The combination was well tolerated. Indeed, DURV + TREM could play a role for the treatment of HCC in patients ineligible for *anti*-VEGF such Bevacizumab due to esophageal varices or elevated bleeding risk. Moreover, ICIs combination could be investigated in combinatory approaches as neoadjuvant, an orphan setting in HCC.

Recently, meta-analyses carried out by Sonbol et al. [43] in 2020, by Liu et al. [44] in 2021 and by Li et al. [45] in 2022 also showed a better performance of ICI + *anti*-VEGF combination. Another recently published meta-analysis by Lei et al. [46] and Fulgenzi C.A.M [47]. showed superior efficacy of ICI + VEGF combination. Our work updated the presently available information by the emerging data on TIS, CAM + RIV, and LEN + PEM, and provide integrated analysis thought Bayesian approach of efficacy and toxicity evaluated in bidimensional plot in order to define in deep the overall scenario.

The different trials analyzed in our work offer new insights in the future therapeutic scenario. Indeed, at present, ATEZO + BEVA appears the best choice in western countries in patients without bleeding risk but some points need to be underilned. Immunotherapy in combination with *anti*-VEGF/VEGFR appears to be main therapeutic option by our results, but no predictive factor of response to immunotherapy has emerged yet by our analysis of included trials and this still a major limitation for treatment planning. However, some potential biomarkers have been investigated [48], so future phase III trials may stratify patients basing on these different factors. Furthermore, the role of immunotherapy in early disease is still unclear, but some recent works [49,50] providethe rationale of using this strategy even in early or locally advanced disease, considering the high ORR emerged by our analysis for immunotherapy combination strategy.

At disease progression, Sorafenib and Lenvatinib could be used as second-line strategy, followed by Cabozantinib or Regorafenib [43] in further treatment lines, if feasible. The main limitation of our study is intrinsic to NMA methodology were, in the absence of direct comparisons, the performance of each treatment is inferred by Bayesian probabilistic methodology. A potential limit of this work is lack of stratification by known prognostic factors as HBV/HCV infection, age or ethnicity, because not enough data could be extracted from the individual trials.

## 5 Conclusion

Our review and meta-analysis offers a comprehensive view of the treatment scenario of the aHCC Indeed, the introduction of Atezolizumab *plus* Bevacizumab is a paradigm-changing approach but since the IMbrave 150 other combinatorial therapies with ICI have been investigated, and in our work the combination of the *anti*-PD-1 Camrelizumab *+* Rivoceranib ranked the best in OS, whereas Lenvatinib *+* Pembrolizumab scored the best in both PFS and Sintilimab *+* Bevacizumab was the best strategy for ORR.

Our study, comparing different therapeutic strategies by the indirect comparisons and network meta-analysis, could help to generate a new sequencing for the treatment of aHCC. Unquestionably, the identification of predictive biomarkers by novel pharmagenomics platforms [[51], [52], [53], [54]] could allow stratification of patient's treatment, which together with a better understanding of the role of the tumor-microenvironment, may significantly help to develop new treatment strategies, improve quality of life and survival of these patients and certainly allow better research in the future.

### Author contribution statement

Domenico Ciliberto: Conceived and designed the experiments; Analyzed and interpreted the data; Wrote the paper. Giulio Caridà: Conceived and designed the experiments; performed the experiments; Analyzed and interpreted the data; Wrote the paper. Nicoletta Staropoli; Caterina Romeo; Grazia Maria Arillotta; Cristina Napoli; Luigia Gervasi; Francesco Luciano, Caterina Riillo: Analyzed and interpreted the data. Pierfrancesco Tassone; Pierosandro Tagliaferri: Contributed, materials, analysis tools or data.

## Data availability statement

Data included in article/supp. material/referenced in article.

## Declaration of competing interest

The authors declare that they have no known competing financial interests or personal relationships that could have appeared to influence the work reported in this paper.
