# Supplementary material for: First-line systemic treatment for hepatocellular carcinoma: A systematic review and network meta-analysis
Source: Heliyon. 2023 Jul 26;9(8):e18696. doi: 10.1016/j.heliyon.2023.e18696 (PMC10407140; doi:10.1016/j.heliyon.2023.e18696)
Supplement: Multimedia component 1 [file mmc1.docx]

**Supplementary S1**

| Pubmed | (((((((((((((((unresectable hepatocellular carcinoma Filters: Clinical Trial, Randomized Controlled Trial) OR (HCC)) AND (immune checkpoint inhibitors)) OR (atezolizumab)) OR (nivolumab)) OR (durvalumab)) OR (tislelizumab)) OR (tremelimumab)) OR (TKI)) OR (sorafenib)) OR (lenvatinib)) OR (cabozantinib)) OR (nintedanib)) OR (linifanib)) OR (bevacizumab)) OR (anti angiogenic therapy)) AND ((clinicaltrial[Filter] OR randomizedcontrolledtrial[Filter]) AND (2008:2022[pdat])) |
| --- | --- |
| Embase | **#2**  **#1** AND (**'atezolizumab'**/dd OR **'bevacizumab'**/dd OR **'cabozantinib'**/dd OR **'doxorubicin'**/dd OR **'durvalumab'**/dd OR **'immune checkpoint inhibitor'**/dd OR **'ipilimumab'**/dd OR **'lenvatinib'**/dd OR **'placebo'**/dd OR **'sintilimab'**/dd OR **'sorafenib'**/dd)  **#1**  (**'advanced hepatocellular carcinoma'**/exp OR **'advanced hepatocellular carcinoma'**) AND ([controlled clinical trial]/lim OR [randomized controlled trial]/lim) AND [2008-2022]/py |
| Cochrane | ID Search Hits  #1 ("hepatocellular carcinoma"):ti,ab,kw OR ("liver neoplasm"):ti,ab,kw OR (HCC):ti,ab,kw OR (liver cancer):ti,ab,kw (Word variations have been searched)  #2 MeSH descriptor: [Liver Neoplasms] explode all trees  #3 MeSH descriptor: [Carcinoma, Hepatocellular] explode all trees  #4 (atezolizumab):ti,ab,kw OR (nivolumab):ti,ab,kw OR (durvalumab):ti,ab,kw OR (tremelimumab):ti,ab,kw (Word variations have been searched)  #5 (checkpoint inhibitors):ti,ab,kw OR (anti-PD1):ti,ab,kw OR (anti-PDL1):ti,ab,kw OR (anti-CTLA4):ti,ab,kw OR ("tyrosine kinase inhibitor"):ti,ab,kw  #6 (sorafenib):ti,ab,kw OR ("sunitinib"):ti,ab,kw OR (brivanib):ti,ab,kw OR (linifanib):ti,ab,kw OR (bevacizumab):ti,ab,kw  #7 ("randomised"):pt OR (randomized):ti,ab,kw OR ("randomised clinical trial"):ti,ab,kw AND ("randomized clinical trial"):ti,ab,kw  #8 #1 OR #2 OR #3  #9 #4 OR #5  #10 #9 OR #6 OR #6  #11 #8 AND #9 AND #10 AND #7 with Publication Year from 2008 to 2022, in Trials (Word variations have been searched) |

**Supplementary S2 – PICOS model**

| Parameter | Inclusion criteria | Exclusion criteria |
| --- | --- | --- |
| Patients | Untreated HCC, first-line | Already treated patients. |
| Interventions | TKI, ICI, anti-VEGF and their combination. | Chemotherapy only, intervention procedures. |
| Comparator | Not applicable. | Not applicable. |
| Outcomes | OS, PFS, ORR, Toxicity. | QoL as only outcome, results not available |
| Study design | Randomized prospective studies. | Not randomized trials, retrospective comparator, not English papers. |

*Inclusion and exclusion criteria according to PICOS model*.

**Supplementary S3 – Risk of Bias summary**


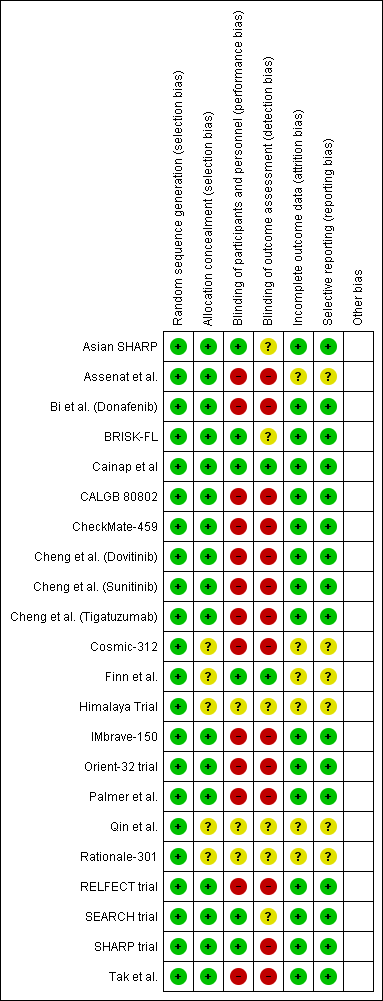


Risk of bias summary according to RoB by Cochrane.

**Supplementary S4 – risk of bias graph**


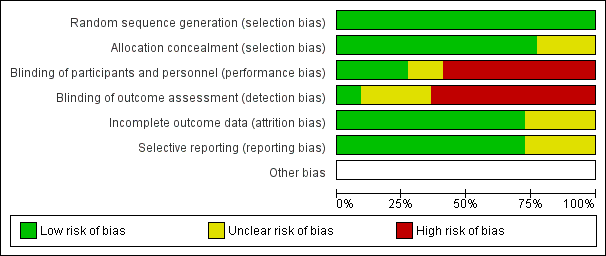


*Risk of bias summary according to RoB by Cochrane*.

**Supplementary S5 – Funnel plots**


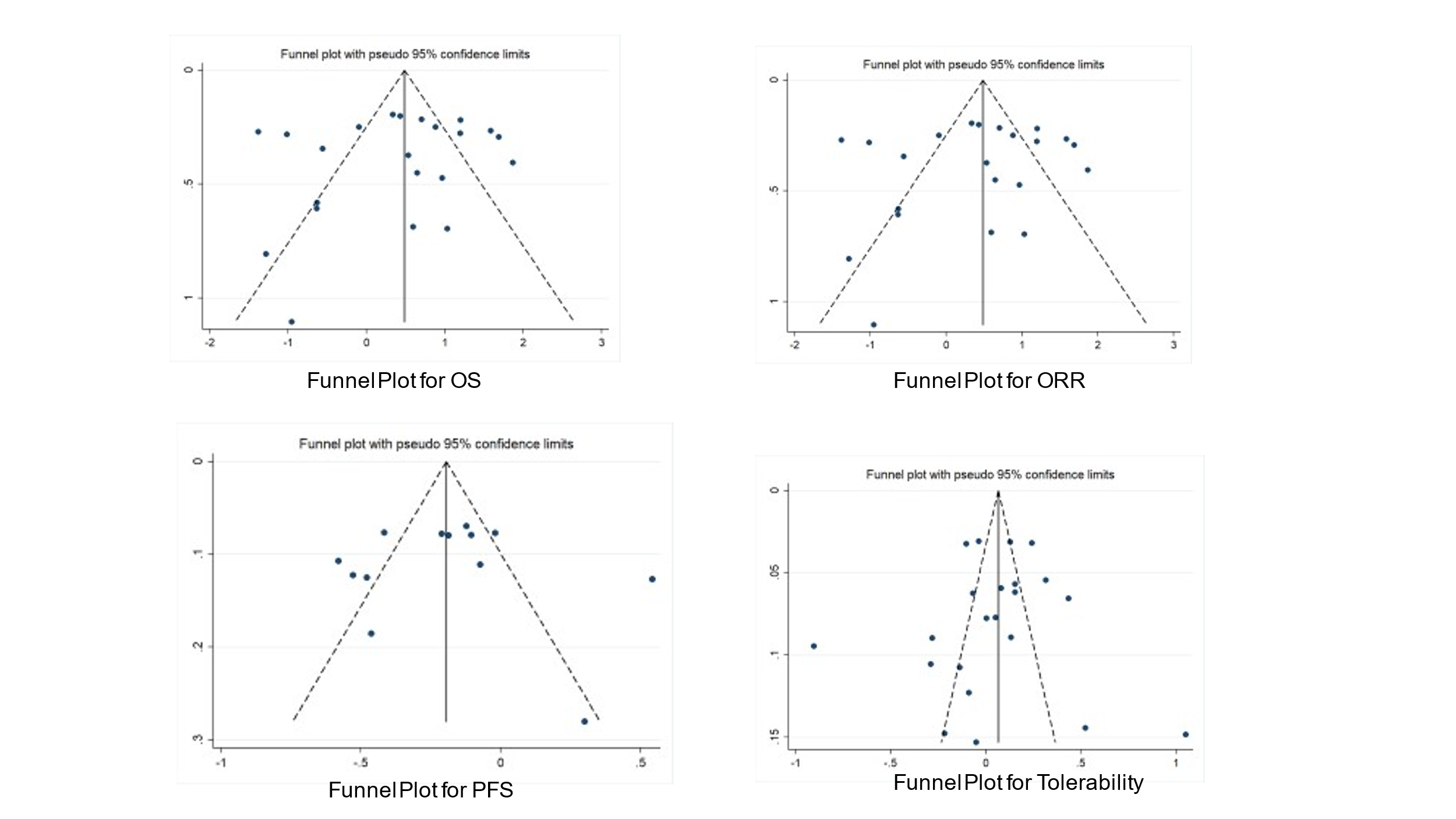


*Funnel plot for each outcome.*

**Supplementary S6 – Networkplot for PFS**


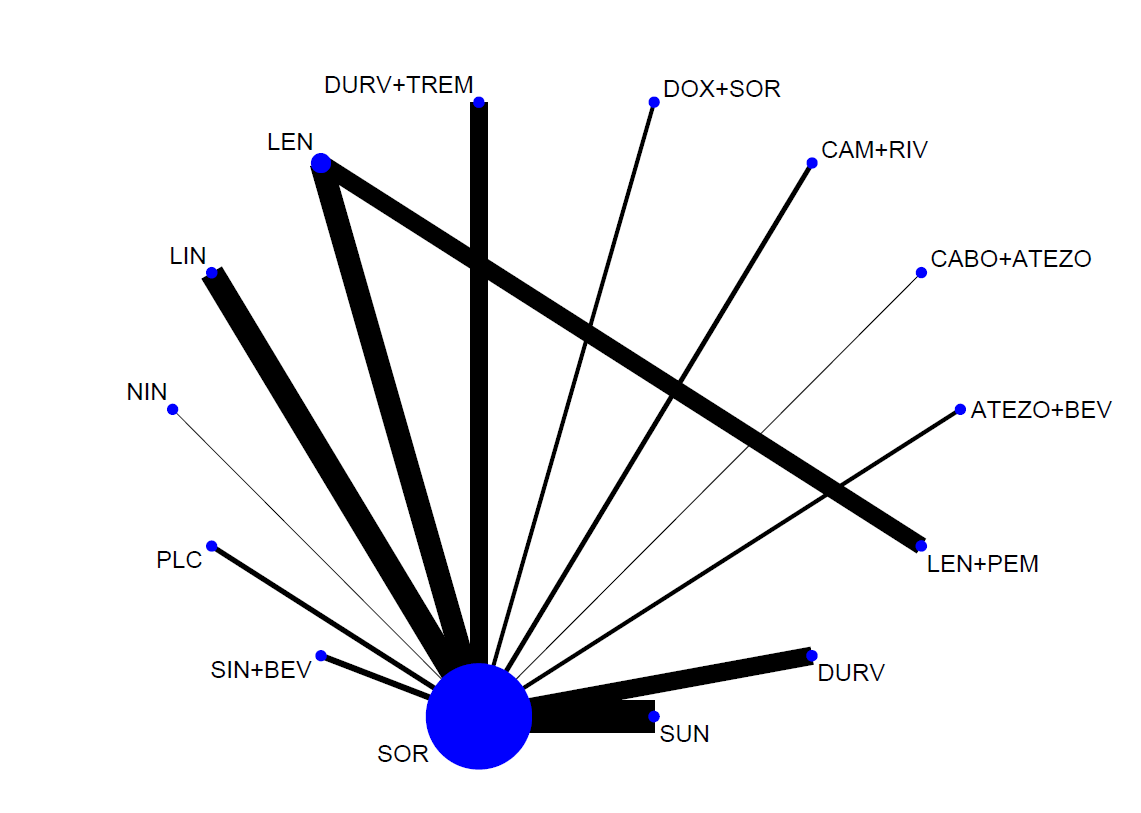


*The image shows the direct comparisons of the network meta-analysis. Each circular node represents a treatment: the larger is the circle, the greater is the number of RCTs in which that treatment was investigated. The thickness of the lines is proportional to the number of patients involved in each comparison.*

**Supplementary S7 – Netleague for PFS**

*This figure shows the estimated summary effect for each comparison in term of HR and relative 95% CrI. The treatments are sorted from the best to the worst according to SUCRAs values. HRs lower than 1 favour the first treatment. If the CrIs contains 1, the result is not statistically significant*

**Supplementary S8 Networkplot for ORR**


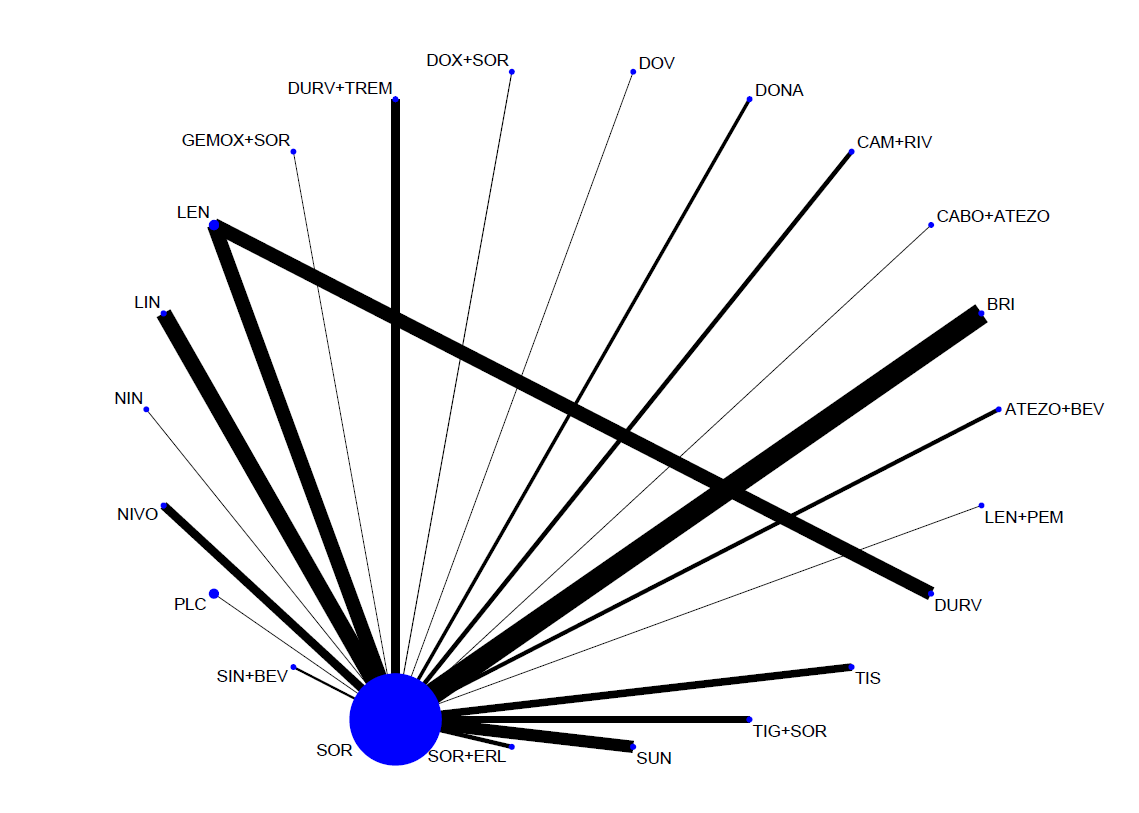


*The image shows the direct comparisons of the network meta-analysis. Each circular node represents a treatment: the larger is the circle, the greater is the number of RCTs in which that treatment was investigated. The thickness of the lines is proportional to the number of patients involved in each comparison.*

**Supplementary S9 – Netleague for ORR**

*This figure shows the estimated summary effect for each comparison in term of Odds Ratio and relative 95% CrI. The treatments are sorted from the best to the worst according to SUCRAs values. Odd Ration lower than 1 favour the first treatment. If the CrIs contains 1, the result is not statistically significant*

**Supplementary S10 – Networkplot for tolerability**


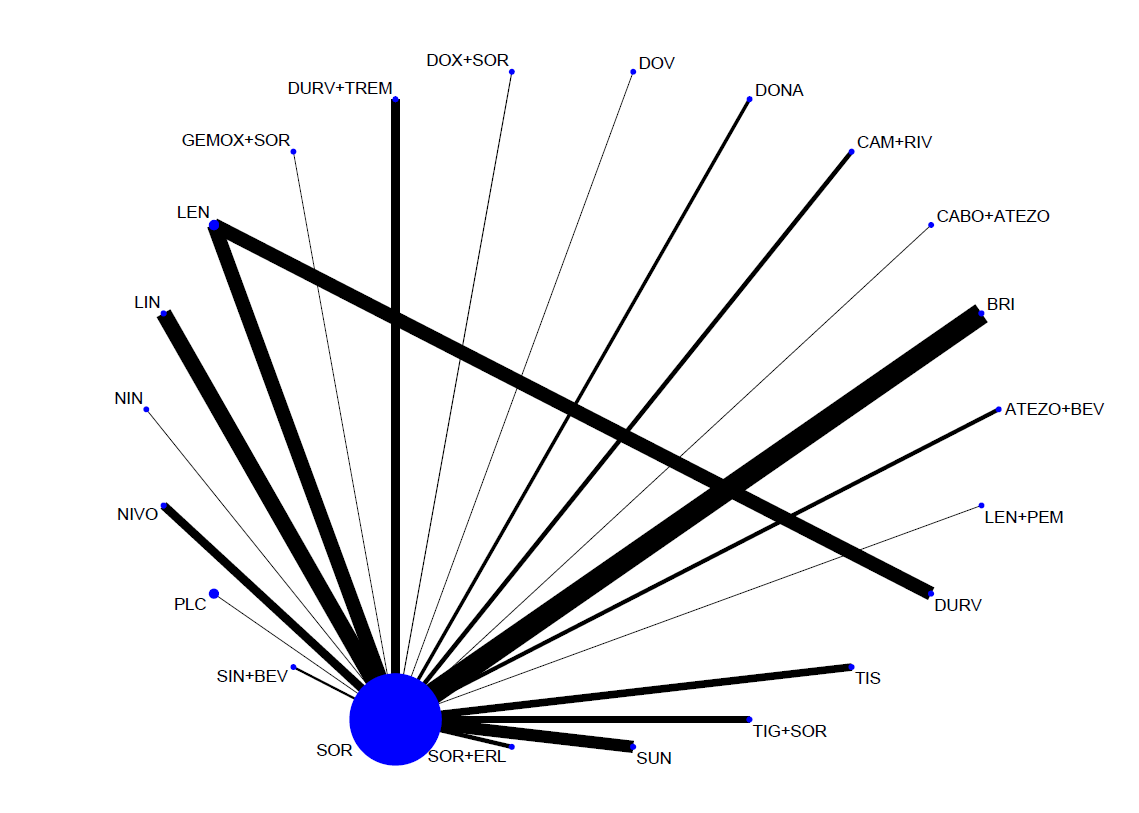


*The image shows the direct comparisons of the network meta-analysis. Each circular node represents a treatment: the larger is the circle, the greater is the number of RCTs in which that treatment was investigated. The thickness of the lines is proportional to the number of patients involved in each comparison.*

**Supplementary S11– Netleague for tolerability**

*This figure shows the estimated summary effect for each comparison in term of RR and relative 95% CrI. The treatments are sorted from the best to the worst according to SUCRAs values. RR lower than 1 favour the first treatment. If the CrIs contains 1, the result is not statistically significant.*

**Supplementary S12 – SUCRAs for OS**


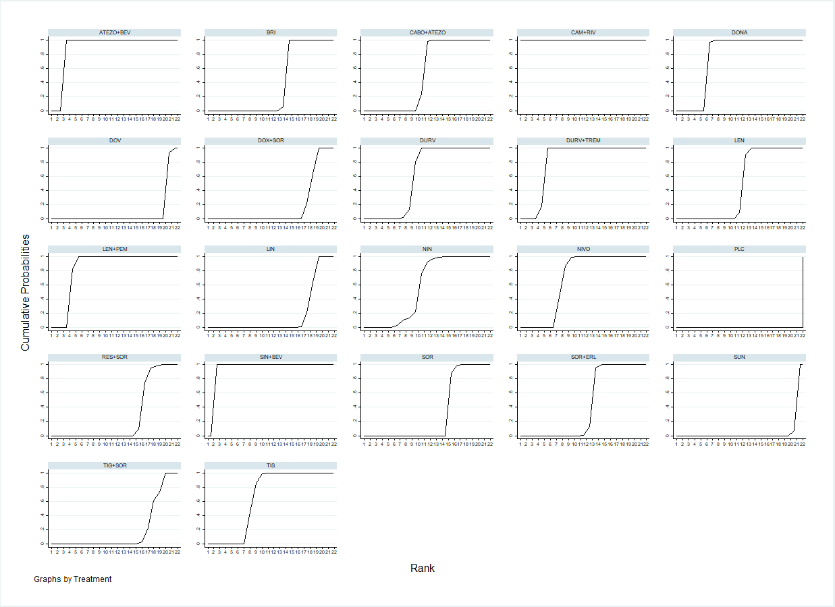


*SUCRA values for OS.*

**Supplementary S13 – SUCRAs for PFS**


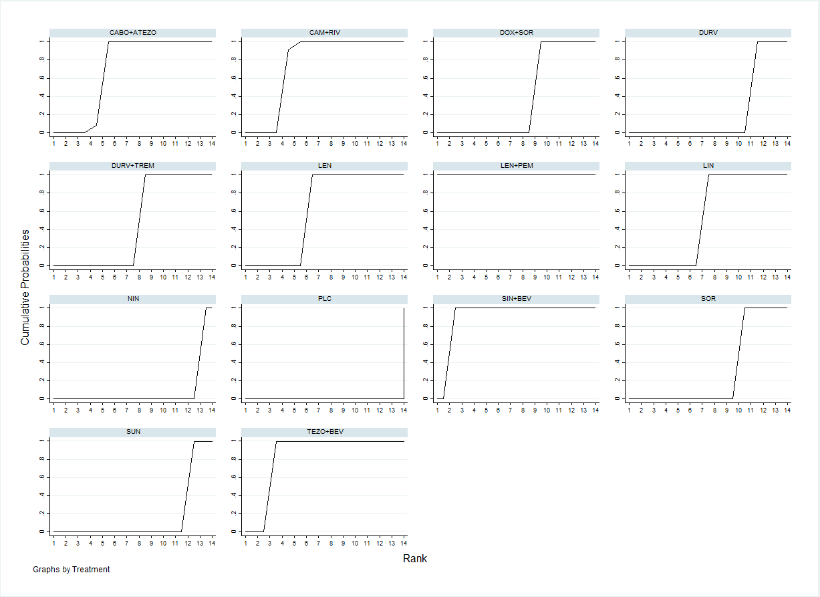


*SUCRA values for PFS.*

**Supplementary S14 – SUCRAs for ORR**


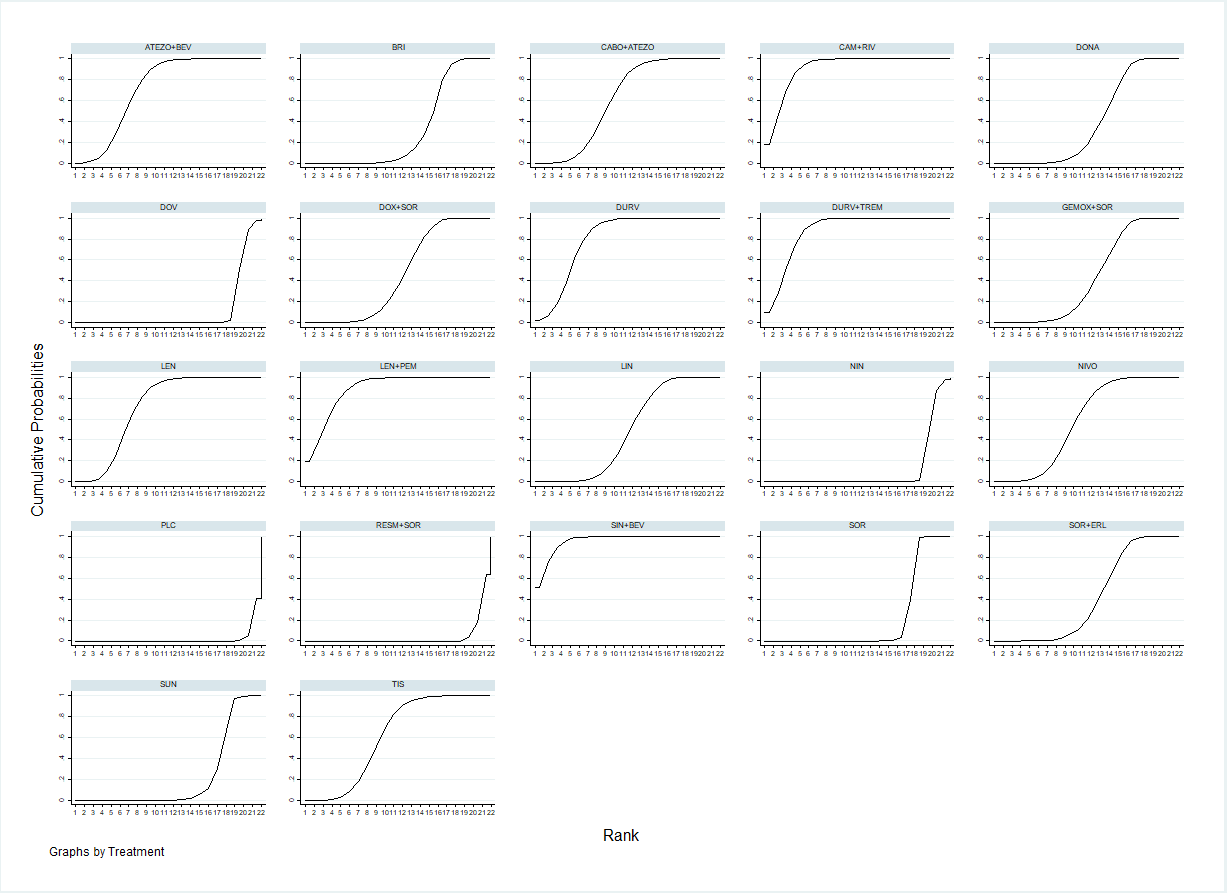


*SUCRA values for ORR.*

**Supplementary S15 – SUCRAs for Tolerability**


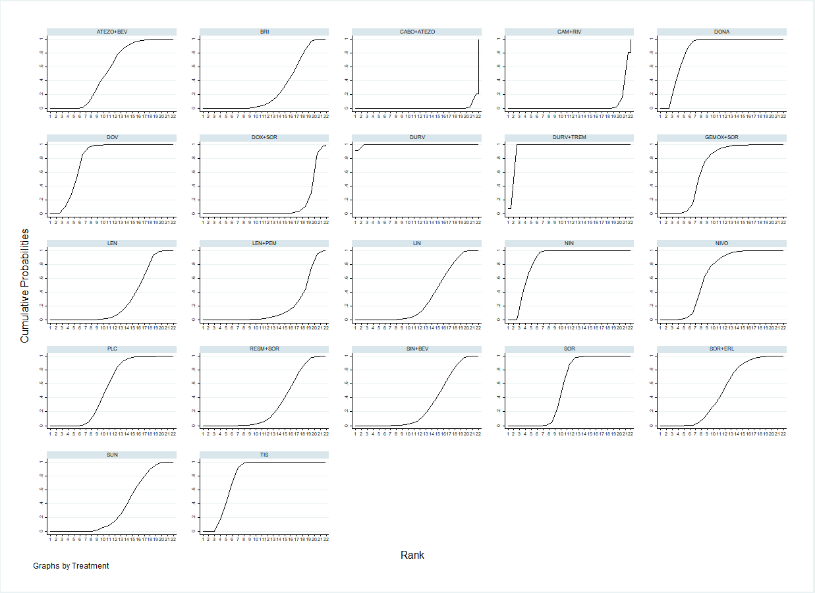


*SUCRA values for tolerability- .*
